# Progressive transformation of germinal centers: an illustration of two clinical cases

**DOI:** 10.1007/s00277-018-3257-1

**Published:** 2018-03-01

**Authors:** Konrad Tałasiewicz, Aleksandra Czachowska, Katarzyna Śmiałek-Kania, Dominika Jaxa-Larecka, Beata Jagielska

**Affiliations:** Department of Oncology Diagnostics, Cardioncology and Palliative Care, Maria Skłodowska-Curie Memorial Cancer Center and Institute of Oncology, Roentgena 5, 02-781 Warsaw, Poland

Dear Editor,

Progressive transformation of germinal centers (PTGC) is a common, but often underdiagnosed cause of benign peripheral lymphadenopathy that is characterized by reactive follicular hyperplasia along with mantle zone lymphocyte expansion into the adjacent sinusoids and germinal centers (GC); the follicles with GC become enlarged and replaced by small lymphocytes from the mantle zone (mainly B cells) [1].

This entity can precede, appear during or after Hodgkin’s lymphoma (HL), mainly nodular lymphocyte predominant Hodgkin’s lymphoma (NLPHL) [2]. The disease itself can also be wrongly diagnosed as early stage NLPHL or follicular lymphoma (FL); therefore, new immunoarchitectural patterns to distinguish between these entities have recently been proposed [1, 2]. We have presented two cases of PTGC along with visual illustrations of the clinical and pathological characteristics.

A 32-year-old male presented with painless submandibular and cervical lymphadenopathy. It is worth noting that the patient was under oncological surveillance after a radical distal pancreatectomy due to neuroendocrine pancreatic cancer pT2N0M0 4 years earlier.

Upon physical examination, we found enlarged lymph nodes level I, II, and III. Laboratory tests were irrelevant, apart from erythrocyte sedimentation rate (ESR) 22 mm/1 h (1–15) and beta-2-microglobulin 1.96 mg/L (0.7–1.8).

**Fig. 1:**
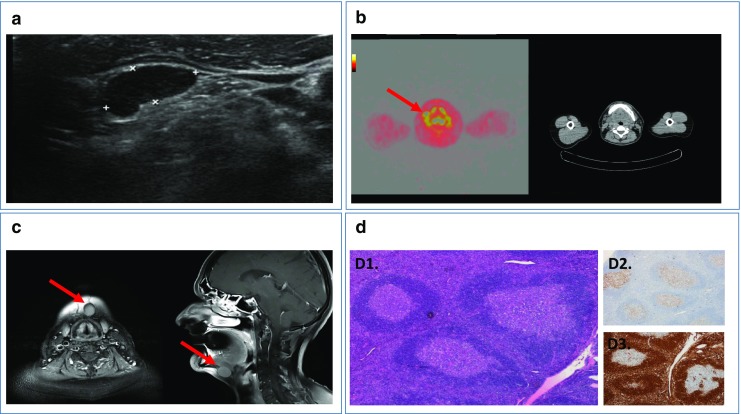
**a** Ultrasound photograph of the hypoechoic lymph node, case 1*.* **b** PET-CT with FDG demonstrating increased uptake of the radiotracer at different levels on the both sides of the neck, case 1*.* **c** MRI showing enlarged submandibular lymph node with contrast enhancement, case 2*.* **d** Histopathology micrographs, D1. H&E staining with follicles of varying sizes and shapes with eccentric mantle layers surrounding their germinal centers, D2. BCL6 staining—normal distribution (BCL6 is noted in a high proportion of Hodgkin’s disease cells), D3. BCL2 staining—normal distribution (mantle zone and T cells are positive but germinal center is negative). It helps distinguishing from follicular lymphoma (germinal centers are BCL2+), case 2

We performed an ultrasound (Fig. 1a), which showed hypoechoic, enlarged (to 24 × 20 mm) lymph nodes without visible hilus, one on which a fine needle aspirational biopsy (FNAB) was done. The results of cytopathology were inconclusive—immunohistochemistry (IHC) staining confirmed LCA (+) and CD56 (−), so our further task was to differentiate between reactive lymphadenopathy and low-grade lymphoma. Due to the patient’s history and a suspicion of lymphoma, a PET-CT with FDG was ordered; it revealed an increased uptake of the radiotracer (SUV_max_ up to 8.9) among many of the cervical lymph nodes (Fig. 1b).

Afterwards, a surgical excisional biopsy was done and showed giant, irregular shaped germinal centers with further IHC that confirmed the diagnosis of PTGC.

A 65-year-old female presented with a single, painless mass in the submandibular region. The patient was under gastroenterological observation due to primary sclerosing cholangitis and silent coeliac disease. Upon examination, the patient had an enlarged, round, and well-shaped submandibular lesion. An otorhinolaryngology examination did not reveal further findings that would suggest the cause of the lymphadenopathy. Laboratory tests were also irrelevant, apart from elevated beta-2-microglobulin 2.02 mg/L (0.7–1.8).

On the ultrasound, we found a hypoechoic lymph node sized 10 × 17 mm with abundant vascularization. A FNAB of the lesion was performed and the cytopathology results suggested a reactive lymphadenopathy and advised a core biopsy in the case of further clinical doubts.

A magnetic resonance was ordered to exclude other pathologic findings in the head and neck area. It showed enlarged submandibular (level IB) lymph node that displayed contrast enhancement (Fig. 1c).

Due to the vague clinical symptoms and the patient’s preference for lesion removal rather than active surveillance, an excisional biopsy was done.

The pathologic report suggested follicular lymphoid hyperplasia with PTGC (Fig. 1D1). The BCL2 (Fig. 1(D3)), BCL6 (Fig. 1(D2)), and CD10 expression was normal.

PTGC is quite a common clinical entity that accounts for 3.5% of cases of chronic lymphadenitis [3]. The disease usually appears on the neck region, affecting predominantly men (3 male, 1 female). The duration of the disease varies—sometimes, it is less than 1 year, but sometimes it can be significantly longer. PTCG is usually found in a single, painless lymph node. It is also worth mentioning that all these symptoms are consistent with NLPHL [4]. PTGC is not considered a premalignant condition although it can precede, coexist or appear after HL. Some studies have demonstrated a connection between the disease with NLPHL [3, 5, 6] although most patients suffer from the disease without any further unfavorable sequelae.

Because of the aforementioned facts, a meticulous pathological differential diagnosis should be determined to exclude such lymphoproliferative disorders like FL or NLPHL.

The link between HL and PTGC suggests that oncological/hematological active surveillance should be proposed. There is no generally acclaimed treatment or surveillance standard for PTGC; however, Picardi et al. suggested that rituximab prophylaxis improve event-free survival in patients with PTGC and a history of HL, which gave us new look into this enigmatic clinical entity which prompted our further studies [7].
